# Nutritional support in patients undergoing haematopoietic stem cell transplantation: a multicentre survey of the Gruppo Italiano Trapianto Midollo Osseo (GITMO) transplant programmes

**DOI:** 10.3332/ecancer.2015.545

**Published:** 2015-06-15

**Authors:** Stefano Botti, Sarah Jayne Liptrott, Gianpaolo Gargiulo, Laura Orlando

**Affiliations:** 1 Complex Operational Unit of Haematology, ASMN-IRCCS Reggio Emilia, viale Risorgimento 80, Reggio Emilia 42123, Italy; 2 Division of Haemato-oncology, European Institute of Oncology, via Ripamonti 435, Milan 20141, Italy; 3 Bone Marrow Transplant Centre, AOU Federico II, via S. Pansini 5, Naples 80131, Italy

**Keywords:** nutritional support, haematopoietic stem cell transplant, malnutrition

## Abstract

A survey within Italian haematopoietic stem cell transplant (HSCT) programmes was performed, in order to obtain a snapshot of nutritional support (NS) in patients undergoing HSCT. The primary objective was to verify whether an evidence-based practice (EBP) approach to NS was implemented in HSCT centres. A multicentre survey was performed by questionnaire, covering the main areas of NS (screening, treatment planning, monitoring, nutritional counselling, and methods of nutritional support). The results indicated a significant variation between clinical practice and evidence-based guidelines in terms of clinical pathways, decision-making, and care provision regarding NS. Further research is required to identify reasons for the limited application of EBP and measures that may be undertaken to address such issues. Development of a multidisciplinary educational programme in order to raise awareness of the issue should be undertaken.

## Introduction

Malnutrition in patients undergoing HSCT is related to a series of factors including the underlying disease, nutritional state pre-transplant, conditioning regimen used, and complications such as graft versus host disease (GvHD) [1]. In HSCT patients, reduced nutritional intake (50–60%) is frequently observed. Also observed are changes in nitrogen balance, energy requirements, glucose tolerance, vitamin absorption, antioxidant requirements, cholesterol, protein, and trace elements [2]. Weight loss and lean body mass may occur as a result of malabsorption, increased catabolism, alterations in biochemical parameters [3, 4], and anorectic effects of cytokines [5]. Furthermore, psycho-emotional factors such as anxiety, depression, and fatigue [6, 7] may also be contributing factors.

**Figure 1.**
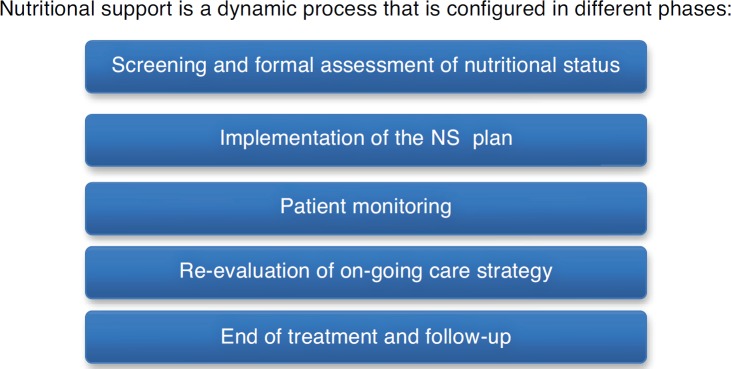
Phases of NS.

Malnutrition can occur rapidly in the absence of NS [8], and may have a severe and negative impact on mortality [9, 10] and morbidity [11, 12], with persistent serious long term effects [13, 14, 15, 16, 12], and hence warrants attention. The international nutritional guidelines and recommendations are targeted towards the avoidance or limitation of the effects of malnutrition and personalising the nutritional intervention. Methods to improve or maintain nutritional intake are known as NS [17], including oral nutritional supplements (ONS), artificial nutrition such as parenteral nutrition (PN), tube-feeding (TF), intravenous administration of nutrients (IVN), and immunonutrition. The main guidelines agree on a multidisciplinary approach [18], that it should involve various stakeholders including patients, doctors, nutritionist/dietitians, pharmacists, and nurses [19] collectively recognised as a Nutrition Support Team (NST). Other complimentary figures i.e. professionals and caregivers, may also be involved at various points during the patients care. NS provided should follow phases (Figure 1), which is documented and regulated by the presence of specific pathways, procedures, or protocols [20].

A nutritional intervention is appropriate in HSCT patients who are malnourished, or expected to have a reduced intake or reduced absorption of nutrients for a prolonged period of time, or have developed moderate or severe GvHD accompanied by a reduced oral intake and/or significant malabsorption [1]. The NST in collaboration with the transplant programme team can deal with the NS process, ensuring that the nutritional intervention becomes a truly integral part of supportive care for the patient [2]. However, HSCT patients are poorly compliant to all that is nutrition and NS [2], and the use of artificial nutritional interventions not only incur numerous side effects but also significantly affect costs [21, 22]. These types of factors including others such as social, psychological, and ethical, can impact on decisions regarding NS provision.

Despite suggestions from the literature, which aim to orient, coordinate, and guide healthcare professionals in providing optimal care, the actual translation from the literature into clinical practice remains challenging, and this may be compounded by a lack of clear evidence for some of the NS methods, where clinical results are often influenced by the complexity of the clinical context.

In order to investigate current clinical practice within GITMO transplant programmes regarding adherence to international literature pertaining to NS, a questionnaire-based survey was created. This was based on the principal phases of the NS process and sent to the nurse representatives of all transplant programmes. The primary objective of this multicentre survey was to verify whether an EBP approach to NS in patients undergoing HSCT existed. This paper presents the results of this survey and also a discussion on implications of this practice.

## Methods

### Participants

The participants were principally nurses with email addresses at GITMO registered centres. Only one respondent was permitted for each centre as the purpose of the survey was to investigate inter-centre variability, and not to investigate intra-centre variability.

### Design

Survey items were drawn from a literature review of key sources (PubMed, Embase, Cochrane Library, National Guideline Clearinghouse, National Institute for Clinical Excellence), in order to identify evidence and recommendations relating to the steps of the nutritional process. Items were reviewed by experts in the field of nutrition, HSCT, and members of the research team. The GITMO Nurses Board piloted the survey in order to refine questions and response options. Formal validity and reliability assessments were not performed. Questions (n = 27) were formatted as multiple-choice and open-ended. The first section collected data relating to the transplant centre itself, and five sections covered the topics:

- Nutrition screening and formal evaluation of patients
- Treatment plan
- Monitoring
- Nutritional counselling
- Methods of support

The questionnaire was placed onto a web platform, and a database was created to facilitate data collection. An invitation email with a link to the survey was sent as well as three reminder mails for those who had not responded. Data was collected from 17 September 2012 to 25 January 2013 using the online web tool. A total of 102 transplant centres were invited and 83 centres responded (81.3%).

Data was collected and downloaded to an excel spreadsheet. This was analysed descriptively by members of the research team.

## Results

Table 1 shows the characteristics of the transplant centres responding. The majority were adult transplant centres, were part of general haematology units, and performed both autologous and allogeneic transplants, with just under one-quarter performing autologous transplant alone.

### Screening and formal evaluation (Table 2) 

Less than half of respondents (n = 36, 43%) reported having formal guidelines/protocols evaluating patient’s nutritional status, 39% of centres did not perform nutritional screening, and 24% only when necessary. Of those centres performing screening (n = 30, 36%), 12% screened at admission, and 24% at admission and regularly until discharge.

A variety of methods were used for screening, including social and dietary information recorded during history taking, use of anthropometric parameters (weight, body mass index, arm circumference, and skin fold), and blood chemistry parameters. Specific nutritional indices were less frequently used (19 centres) and only five centres used nutritional tools. In the majority of cases, screening was performed by healthcare professionals from within the transplant team (doctors in 32 centres and nurses in 27 centres) whilst professionals such as nutritionists (11 centres) and dietitians (13 centres), were less frequently involved.

### Treatment plan

Most centres had formal treatment plans for nutritional care (n = 42, 51%), yet ‘who’ was involved in decision-making regarding NS varied. Almost three-quarters of centres (n = 59, 71%) reported the presence of either a nutritionist or a nutrition team, yet 23% of centres lacked a professional to refer to for decisions regarding NS therapies. Despite the high presence of nutritional experts, creating conditions for multidisciplinary team working, just 22% of centres (n = 18) involved all personnel in the decision-making process. Most decisions were made within the transplant team by either the haematologist 26% (n = 22), or the haematologist and nurse 30% (n = 25). In 18% of centres (n = 15), decisions were made by the nutritionist in collaboration with the haematologist, and in 4% of centres (n = 3) by the nutritionist alone.

### Monitoring

In 65% of centres, there was no standardised protocol/procedure for monitoring nutritional status, and 40% of centres did not monitor or document oral food intake. Forty-five per cent of respondents reported other methods of monitoring e.g. recognition of anthropometric (27 centres) and/or biochemical parameters (29 centres), with less frequent use of specific nutritional indices (12 centres), and nutrition specific tools (three centres). These parameters were recorded with varied frequency, with only 25% of centres reporting daily recordings and the remaining 13% at least weekly. Sixty-two per cent of centres responded ‘other’, not providing any further detail. With regards to follow-up after discharge, no further monitoring was the case in 48% of centres, whilst 21% reported always monitoring nutritional status, and in 31% of centres, this continued only where patients had experienced nutritional difficulties during hospital admission.

### Nutritional counselling

In 78% of centres, issues relating to nutrition and support were discussed in the pre-admission transplant discussion, however only 59% of centres provided written information for patients and family. During hospitalisation, nutritional counselling was performed in 23% of centres, but not in 24%, with the majority of centres (53%) providing counselling only if necessary. Counselling was usually performed by staff from the transplant team (doctors n = 36, or nurses n = 28), but less frequently by other professionals such as the nutritionist (29 centres).

When asked about the timing of nutritional counselling, 63 centres performed this at admission if necessary, 16 centres during the pre-admission transplant discussion, 13 centres at admission, and only 6 centres performed this routinely. Forty-five percent of centres specified that there were no conditions where the opinion of patients and family members would be sought, whereas 48% of centres said there were conditions–without further detail given, and 7% said that written consent was obtained.

### Methods of NS (Table 3)

Diverse methods of NS were used, with PN being the most widely used technique (76 centres), followed by the use of ONS (47 centres), and IVN (33 centres). A limited number of centres used enhanced oral diet (OD) with food fortification (FF) (23 centres) and nasogastric enteric methods (TF) in nine centres. It was observed that only 7/83 centres considered the full range of artificial nutritional options available.

Three-quarters of centres (75%) did not use immunonutrients, however 13% stated using glutamine, 8% eicosapentaenoic acid, or ‘other’ nutrients (4%). As expected, 80% of centres did not consider TF, whilst 14% of centres used this technique only in specific clinical conditions that do not always coincide with literature indications of its use. Almost half of responding centres used ONS, and of these 19% only in specific clinical conditions, which were not otherwise specified.

Seventy-nine per cent of centres reported use of a predominantly ‘standard oral low microbial diet’. Conflicting information arose where no transplant centre reported that patients are refused OD, yet in 10% of centres an OD is not permitted in some phases of treatment. Where NS therapy was necessary, 70% of centres opted for PN as first line, 13% gave IVN, 9% ONS, 7% FF, and in 1% of centres TF.

## Discussion

In order to construct adequate plans for nutritional assistance, hospital organisations should adopt specific policies and protocols in order to identify patients at nutritional risk, undertake nutritional screening, support, assistance, and document care [19, 20], yet this survey reported less than half of respondents had protocols in place. Such documents contribute to patient compliance, appropriateness of treatment, improvement in nutritional outcomes, and at the same time, reduce costs of PN as well as interdisciplinary conflict [23], which can be a source of difficulty in clear and accurate care planning. Multidisciplinary working is fundamental in all phases of the patients’ pathway. Although the concept of the NS team is well recognised, it is not always present in practice [19]. Nearly one-quarter of centres had no professional figure to refer to for decisions regarding NS, and often all multidisciplinary team members did not make the decisions regarding nutritional care, as the literature would suggest [19], and often specialist professionals such as dietitians and nutritionists tended to only be marginally involved. The substantial lack of specific pathways and shared care approaches suggests a problem in clarity of the issue itself, potentially leading to a lack of adherence to and interpretation of the literature.

It is known that patients undergoing myeloablative HSCT are at risk of malnutrition and hence should be screened to identify those requiring formal assessment and development of a nutritional care plan [1], however, screening was not routine practice in the majority of centres and often tools used for screening were non-specific parameters. Screening should indicate the body mass index and unintentional weight loss, considering the length of time, and possibility of future reduced nutrient intake. The Malnutrition Universal Screening Tool (MUST) [24], for example, can facilitate this [17]. Similarly, despite literature supporting the use of protocols for nutritional, anthropometric, and clinical monitoring for NS in hospital [17], the survey revealed just over one-third of centres had such protocols available, whilst others reported some monitoring activity that did not follow a clear or logical pattern. Monitoring protocols facilitating recording of oral intake such as that identified in the National Institute for Clinical Excellence (NICE) guidelines for malnourished patients, provide a basis for practice and aid documentation and evaluation of nutritional status.

In this survey, patients were reported to have a predominantly ‘standard oral low microbial diet’, which itself can be complex for patients to understand, i.e. what they can/cannot eat and when to eat. It is acknowledged that patients should receive dietary counselling regarding ‘risk’ foods that are potential sources of infection, and food preparation in a hygienic and safe manner during the period of neutropaenia [1]. The results suggested that specific nutritional counselling is poorly structured and often implemented ‘as required’, with few centres indicating the involvement of professionals other than nurses and doctors in counselling, for which an insufficient collaboration with dietitians, nutritionists, and psychologists was noted. The benefits of intensive and personalised dietary counselling have showed positive effects on weight maintenance, energy level, and protein intake [25], and a statistically significant benefit on some aspects of quality of life in malnourished oncology patients [26], yet it was not regularly applied by centres responding in this survey.

Methods of NS remain varied and the question may be asked whether choices made are based on familiarity with certain products rather than evidence-based practice. In contrast to the suggestions from the literature over the last ten years where an enteral approach is preferred, at least initially [27], the survey findings showed the use of PN and IV nutritional routes. Traditionally, PN has been the first option for NS in transplant recipients, yet in recent years, it has been gradually reduced in favour of enteral nutrition (EN) [4]. Some authors suggest that the use of PN in HSCT should be reserved as a rescue option, possibly in association with enteral route administration [28].

With regards to immunonutrients, research suggests that patients undergoing HSCT may benefit from pharmacological doses of parenteral glutamine [1, 29, 30] and some suggestions exist about use of eicosapentaenoic acid (ω-3) [31, 32], however less than one-quarter of centres stated using immunonutrients, perhaps because of limited familiarity with such aspects of NS. Similarly, despite the use of ONS in malnourished patients with cancer being shown to be beneficial in terms of maintaining body weight, reducing complications, and mortality [17], over one-half of respondents did not use ONS in their centre. ONS should be considered to improve the nutritional intake in people who can swallow safely, but are malnourished or at risk of malnourishment. They require counselling prior to initiating support, prescription, screening and nutritional outcome monitoring [17]. Despite the findings relating to prohibition of OD in HSCT patients in some treatment phases, the questionnaire did not collect data relating to the phases of the transplant in which OD was prohibited. However, the data remains a concern for the known sequelae and impact on outcomes that lack of oral intake in transplant patients can cause [33, 34].

## Conclusion

We report the results of a national survey of transplant centres looking at nutritional care for HSCT patients, in which a notable variation in practice was observed. One reason may be related to the weighing of the recommendations, as these are the result of complex analyses, which are often poorly supported by the presence of high quality evidence. This can cause objective difficulties in conducting studies in this context, however, this cannot constitute a valid reason to continue with practices that appear in the literature to have been superseded.

The discrepancy between guidelines/recommendations and current practice merits further review, as the findings from this study are limited to a specific cohort of patients, a single country, and perceptions of a single group of healthcare professionals. Future studies incorporating both a larger sample and a variety of healthcare professionals and countries, would provide information as to whether these data are consistent with a wider setting, whilst also attempting to compare and contrast multidisciplinary perspectives on this issue. Although, HSCT is somewhat unique in certain aspects of clinical care and its potential complications, the need for nutritional evaluation and support is a theme not only for HSCT patients. With this in mind, an evaluation of approaches to nutritional care issues in other healthcare settings and pathologies may be advantageous, not only in identifying common or differing topics, but also perhaps in providing examples of best practices. Future studies should also expand on the data from this survey in order to identify barriers to transferring evidence into practice and what could facilitate this process.

In many cases NS appears to be a reactive approach addressing the problem when it clinically manifests, rather than employing a proactive approach. Scarce informative material for patients and caregivers, their involvement in treatment options, and pathways for nutritional follow-up further evidence this. The development of a multidisciplinary educational programme in order to raise awareness of this issue should be undertaken, with the aim to facilitate acquisition of knowledge in this field, and promote a multidisciplinary approach to NS by providing a basis for disseminating evidence. This could cover all aspects of the NS process and include practical aspects such as procedure writing in order to facilitate protocol/policy development, and ultimately enhance patient care.

As a starting point, GITMO Nurses Board has initiated a nutritional awareness and educational programme for nurse members, which is being conducted over a three year period (2014–2016). The programme includes: development and sharing of an evidence-based guidance on nutrition support (unpublished material), insertion of specific sessions in all GITMO conferences, workshop meetings, and training courses. A repeat of this survey is planned for the end of the training course to evaluate educational programme efficacy on nutrition issue awareness and practice.

## Conflicts of interest

Authors declare no conflicts of interest.

## Figures and Tables

**Table 1.** Transplant centre characteristics.

	Responses (%)
Patient group	
–Adult only	61 (73%)
–Paediatric only	15 (18%)
–Both adult and paediatric	7 (9%)
Centre organisation	
–SCT unit	33 (40%)
–SCT withinhaematology unit	50 (60%)
Transplant type performed	
–autologous, allogeneic, and MUD	55 (66%)
–autologous and sibling allogeneic	9 (11%)
–autologous	19 (23%)

MUD—Matched Unrelated Donor SCT—Stem cell transplant

**Table 2.** Screening and formal evaluation.

Topic (question)	Results	n (%)
Screening and formal evaluation		
*In your centre, is there a guideline, protocol, or procedure for the evaluation of the patients nutritional status?*	Yes No	36 (43%) 47 (57%)
*In your centre, is a screening assessment performed to evaluate nutritional status and risk?*	No screening Screening only if necessary Screening at admission only Screening at admission and regularly throughout inpatient stay Other (not specified)	32 (39%) 20 (24%) 10 (12%) 20 (24%) 1 (1%)
*In centres where screening took place, how did this take place (more than one response possible):*	Part of history taking (social and dietary) Anthropological parameters Blood chemistry parameters Specific nutritional indices Specific nutritional tools Other (not specified)	37 34 34 19 5 1
*In centres where screening took place, who performed the screening:*	Haematology doctors Nurses Nutritionists Dietitians Other (Specified: Head Nurse)	32 27 11 13 1

**Table 3.** Methods of NS.

Topic (question)	Results	n (%)
**Methods of NS**		
What methods of NS are used in your centre? *(more than one response possible):*	PN ONS IVN FF TF All methods Other (not specified)	76 47 33 23 9 7 1
Do you use immunonutrients?	No Yes—glutamine Yes—eicosapentaenoic acid (ω3) Other (not specified)	62 (75%) 11 (13%) 7 (8%) 3 (4%)
Do you use tube feeding for HSCT patients?	No Only in specific clinical conditions Yes	66 (80%) 12 (14%) 5 (6%)
Do you use ONS with HSCT patients?	Yes No Only in specific clinical conditions	25 (30%) 42 (51%) 16 (19%)
In your centre, what kind of OD is normally given to patients?	LBD OD is prohibited in some stages of treatment LBD + ONS/immunonutrients LBD + FF OD prohibited Other (not specified)	66 (79%) 8 (10%) 4 (5%) 3 (4%) 0 (0%) 2 (2%)
In your centre, for patients requiring NS, what is your first line approach?	PN IVN ONS FF TF Other	58 (70%) 11 (13%) 7 (9%) 6 (7%) 1 (1%) 0 (0%)

FF: (Food Fortification – enhanced oral diet), IVN: (Intravenous administration of nutrients), LBD: (Standard Low bacterial Diet), OD: (Oral Diet), ONS: (Oral nutritionnel suppléments), PN: (Parenteral Nutrition), TF: (Tube Feeding/Nasogastric enteric methods).
